# Epidemiology, outcomes, and risk factors of traumatic brain injury in Bangladesh: a prospective cohort study with a focus on road traffic injury-related vulnerability

**DOI:** 10.3389/fpubh.2025.1514011

**Published:** 2025-02-14

**Authors:** Farah Naz Rahman, Sukriti Das, Manzur Kader, Saidur Rahman Mashreky

**Affiliations:** ^1^International Centre for Diarrhoeal Disease Research Bangladesh (icddr,b), Dhaka, Bangladesh; ^2^School of Public Health and Preventive Medicine, Monash University, Melbourne, VIC, Australia; ^3^Department of Neurosurgery, Bangabandhu Sheikh Mujib Medical University, Dhaka, Bangladesh; ^4^Department of Medical Science, School of Health and Welfare, Dalarna University, Falun, Sweden; ^5^Centre for Injury Prevention and Research Bangladesh (CIPRB), Dhaka, Bangladesh; ^6^Department of Public Health, North South University, Dhaka, Bangladesh

**Keywords:** traumatic brain injury, road traffic injury, epidemiology, risk factors, health management, Bangladesh, LMIC

## Abstract

**Background:**

Low-and middle-income countries bear a disproportionate burden of traumatic brain injury (TBI), with significant consequences for affected individuals and health systems. However, evidence on the incidence, risk factors, and associated vulnerability—particularly from road traffic injuries (RTIs)—remains limited in South Asia, including Bangladesh, which has one of the highest RTI burdens globally. This study aimed to investigate the epidemiology, clinical characteristics, health outcomes of TBI, and the vulnerability and risk factors associated with RTI-related TBI in Bangladesh.

**Methods:**

A prospective observational cohort study was conducted at Dhaka Medical College and Hospital from May to June 2017. Data were collected during patient admission and at 30-day follow-up (or discharge). Registered medical practitioners used a semi-structured questionnaire to collect data, including the Glasgow Coma Scale (GCS), Glasgow Outcome Scale (GOS), and EuroQol-5D-3L. Descriptive analysis was used to present the incidence, clinical characteristics, outcomes, and pre-hospital care scenarios for TBI. The Chi-square test and multivariable logistic regression analysis were performed to identify the vulnerability of RTI-related TBI and its predictive factors for mortality.

**Results:**

The study followed 659 TBI cases. The mean age was 32.3 years, and the majority (80.1%) were male. RTIs were the leading cause of TBI (62%), followed by physical assault (17%) and falls (16%). The mortality rate was 10.3% (*n* = 68). Patients with RTI-related TBI had 1.95 times higher odds (95% CI 1.21–3.14) of severe GCS scores (<8) and three times higher odds (95% CI 1.59–5.78) of mortality compared to other causes. Predictive factors for mortality included severe GCS (<8) (aOR 8.1, *p* < 0.01, 95% CI 4.1–16.2), loss of consciousness >1 h (aOR 4.4, *p* < 0.01, 95% CI 1.4–8.1), and treatment initiation delay >8 h (aOR 2.8, *p* < 0.01, 95% CI 1.4–5.5). Nearly all patients lacked first aid and emergency transport, and two-thirds were referred from primary and secondary facilities, with one-third receiving no treatment before referral.

**Conclusion:**

RTI is the most vulnerables, duration of unconsciousness, and treatment delays are key predictors of mortality. These findings can inform policy for prevention and management of TBI in Bangladesh and similar settings.

## 1 Introduction

Traumatic brain injury (TBI) is an acute event, occurred when an external mechanical force disrupts the normal function of the brain; usually results from a violent blow or jolt to the head or any object penetrating the skull (1). As estimated in 2019, 27 million people worldwide experience TBI in a year (2). The proportion of TBI cases is almost 3 times higher in low-and-middle-income countries (LMICs) than high-income countries (HICs) (2). With this high incidence rate, it emerged as the leading cause of death and disability due to any traumatic events (3). TBI not only bears grave physical consequences, but it also has a substantial impact on the mental health condition of the patients. It is responsible for the highest prevalence of cognitive and behavioral disorders among young adults as well as long-term neurobehavioral disability in many survivors (4, 5). Furthermore, TBI causes significant social and economic burden to the affected family as well. Due to disability, long-term complications, cognitive and behavioral dysfunction, a large number of patients find it difficult to reintegrate to the employment sector and resume normal social life (6, 7). In addition to the individual economic crisis, TBI consumes a considerable amount of health care costs and resources of a country (8). TBI accounts for 7 million Years Lived with Disability (YLDs) and was estimated to impose an annual economic burden of approximately 300 million USD in North America as of 2009 (2, 9).

Road traffic injury (RTI) is identified as the major cause of TBI by most of the studies (10–13). A review article on the global incidence of TBI reported that LMIC’s have the greatest proportion of TBI resulting from road traffic collisions (14). The study also shared that Southeast Asia and Africa region has the highest burden of RTI related TBI as more than half of the cases of TBI results from road traffic crashes in these regions (14). Globally, Africa has the highest rate of road fatalities followed by Southeast Asia region (15), which often results from head injuries occurred in the process. RTI was reported to be responsible for around 60% of the TBI incidents and mortality in a hospital-based study in Sub-Saharan Africa (16). A couple of hospital-based research in India has also identified RTI as the major cause of TBI incidents, and related morbidity and mortality as a consequence (17, 18). Bangladesh shares a similar scenario where RTI plays a major role in causing TBI as well (19, 20). Due to the increasing incidence of mortality among TBI resulting from road traffic crashes, an effective and prompt pre-hospital care is vital in this regard (10, 18). Studies have shown that a comprehensive post-crash response system, including rapid resuscitation and transport, can significantly improve the outcome and reduce TBI related mortality in patients (10, 21).

Bangladesh is experiencing a surge in RTI. According to WHO Global Status Report on Road Safety, every day 68 people die due to road crashes in Bangladesh (22). It subsequently causes a substantial amount of brain injuries, the combination of which is one of the most vulnerable for mortality among all types of injuries. It also mostly affects the young age group (13, 19) and thus incapacitate a large portion of the productive population of the nation. Frequent anecdotal evidence is found in the newspapers and electronic media that focus on individual incidents of RTI in Bangladesh portraying inadequate pre-hospital care and transport delivery system. As the adequate post-crash response is vital in reducing the mortality of RTI related TBI, generating empirical research-based evidence in this regard is an urgent need.

TBI also disproportionately affects low-and middle-income countries (LMICs), where its consequences place a significant burden on healthcare systems due to limited infrastructure and treatment availability (23). These constraints often contribute to poorer outcomes for TBI patients in LMICs, where TBI-related mortality rates are three to four times higher than in high-income countries (24). An evidence synthesis on TBI management in LMICs has highlighted the urgent need for more studies to evaluate the effectiveness and suitability of Brain Trauma Foundation (BTF) guidelines in low-resource settings (23). Additionally, the challenge of limited pre- and post-hospital care must be addressed when developing strategies for TBI management (23, 24). In LMICs, social determinants of health—factors influencing an individual’s environment, socioeconomic status, and access to care—are closely associated with poorer outcomes, underscoring the need to address these determinants to close the care delivery gap (23). However, data on TBI from LMICs, especially in South Asia, remains scarce (23, 24). In Bangladesh, while some studies have provided insights into the burden and patterns of TBI, identifying road traffic injury (RTI) as the leading cause, only one cross-sectional study has explored factors affecting prognosis (25–27). There is a notable lack of information on the pre-hospital care landscape and its impact on outcomes, as well as on the clinical factors influencing recovery among TBI patients. This calls for more research in Bangladesh to establish effective management guidelines that consider both pre-hospital and clinical factors, to improve patient outcomes and address the unique challenges of TBI management in resource-limited settings.

Against this backdrop, this study was conducted to explore the epidemiology and clinical characteristics of traumatic brain injury at the largest teaching hospital of Bangladesh, and to determine the vulnerability of RTI related TBI along with the clinical and pre-hospital care factors that influence its outcome. Specifically, the study aimed to describe the epidemiology of TBI by examining the causes of injury, and post-injury response with pre-hospital care. Post-injury response with pre-hospital care was assessed in terms of receiving first aid, the location where treatment was first sought and received, and the time gap between injury and treatment initiation. The clinical characteristics of TBI were detailed by evaluating patients’ conditions at admission and during follow-up, using structured scales to measure key clinical indicators along with the status and duration of loss of consciousness and post-traumatic amnesia. The study also sought to evaluate the health outcomes of patients with TBI, focusing on major outcomes such as mortality and health-related quality of life. Furthermore, the research examined the vulnerability of RTI-related TBI by comparing the clinical conditions and outcomes of these cases with those of TBI resulting from other causes. Factors influencing the outcomes of RTI-related TBI were also assessed to provide a comprehensive evidence-based insights for developing policy and programmatic actions aimed at preventing TBI in Bangladesh.

## 2 Methods and materials

### 2.1 Study design, site, and population

A prospective observational cohort study was conducted at Dhaka Medical College and Hospital (DMCH), a government-run tertiary healthcare facility and the largest teaching hospital in Bangladesh, serving patients nationwide. The study enrolled all patients with a primary diagnosis of Traumatic Brain Injury (TBI) who received treatment at DMCH and provided consent to participate, during the period from May 1st to June 30th, 2017.

### 2.2 Case ascertainment

Traumatic Brain Injury (TBI) was defined according to the International Classification of Diseases, 10th Revision (ICD-10). Eligibility was determined based on the diagnosis made by the initial attending clinician. All eligible patients who provided consent were enrolled in the study and followed up. TBI was considered a nature-of-injury, with data collected on its causes and outcomes. The patient’s clinical condition at both admission and follow-up, including any instances of clinical death, was documented using the attending clinician’s declaration in the hospital medical records, as well as assessments by the Glasgow Coma Scale (GCS), Glasgow Outcome Scale (GOS), and EuroQol-5D-3L (EQ-5D-3L).

### 2.3 Data collection method and instrument

Two registered medical practitioners were engaged to collect data through face-to-face interviews with patients or, if the patient was unable to respond, with their attendants. Data collection occurred at two points: upon admission and either at discharge or 30 days post-admission, whichever came first. The choice of a 30-day follow-up period aligns with its widespread use as a benchmark for evaluating early outcomes, including mortality, neurological recovery, and functional status in clinical studies, including TBI (28). The International Mission for Prognosis and Analysis of Clinical Trials in TBI (IMPACT) database also supports using 14- and 30-day mortality as critical endpoints for outcome prediction, reinforcing the significance of the 30-day timeframe in TBI research.

Data was collected using a pre-tested, semi-structured questionnaire, which included sections designed to gather information on the patients’ socio-demographic characteristics, epidemiological details of the injury, pre-hospital management, clinical presentation at admission, and health outcomes during the follow-up. The questionnaire included the Glasgow Coma Scale (GCS) and the Glasgow Outcome Scale (GOS), both of which are widely recognized as reliable tools for assessing the clinical status and outcomes of patients with TBI.

GCS score was used at the time of admission to assess the severity of the patients’ clinical condition. GCS has a score level from 3 to 15, where 3 represents most severe and 15 represents the normal condition (29). The score is further divided into three levels of severity where score 3–8 is considered as the severe category, 9–12 as moderate category and 13–15 as mild category (29). At the time of follow-up, GOS and EuroQol 5 domains 3 levels questionnaire (EQ-5D-3L) were used to measure the health outcome of the patients. GOS has a five scale measurement from scale-1: death to scale-5: good recovery (resumption to normal life) (30). The middle scales are, scale-2: the patient is in a coma or vegetative state, scale-3: the patient is dependent on others for daily life activities and scale-4: the patient can perform daily activities independently but has a minor neurological or psychological deficit (30). EQ-5D-3L is a respondent-reported standardized measurement of health status. This study used the descriptive system of EQ-5D-3L that measures the health condition of the patients in five domains (Mobility, self-care, usual activities, pain/discomfort, anxiety/depression), and has three stages of responses (no problem, some problem, extreme problem) for each domain (31).

### 2.4 Data analysis

Descriptive analysis was done to identify the socio-demographic characteristics of the respondents, cause and characteristics of the injury, and the pre-hospital care factors. The vulnerability of RTI-related TBI in comparison to TBI due to other causes was measured in terms of the severity of the injury and its consequences. Chi-square test was performed and Odds Ratios (OR) were calculated. The outcome variable was whether the injury mechanism was RTI or not. To compare the difference in the severity of the injury, variables presenting clinical condition at the time of admission were included, which are GCS score, duration of loss of consciousness and post-traumatic amnesia, and associated injuries. Mortality outcome, GOS outcome and EQ-5D-3L outcome were analyzed to compare the difference in health outcome between RTI and other injury mechanisms. Both chi-square test and multivariable logistic regression were performed and OR were calculated to identify the factors playing a role in RTI-related TBI mortality. Socio-demographic, clinical and pre-hospital care variables were included in the chi-square test to identify the association with mortality. Socio-demographic factors included sex, education and family income; clinical variables included GCS score, duration of loss of consciousness, duration of post-traumatic amnesia and associated injury; and pre-hospital care variables included time gap in treatment initiation and use of safety measures. Further, multiple logistic regression was done to identify the predictive variables for mortality in RTI-related TBI. Age, sex, education and income were included as part of socio-demographic variables in the regression. The regression also included GCS, duration of loss of consciousness, and associated injury as clinical variables and time gap in treatment initiation as the pre-hospital care factor. During regression, the adjusted odds ratio (OR) was calculated by adjusting the impact of all covariates. For statistical analysis, SPSS v24 was used, and a 5% significance level was considered.

## 3 Results

During the recruitment period, 719 eligible TBI cases were identified at the time of admission, and 659 patients consented to participate in the study, resulting in a response rate of 91.7%. All enrolled participants who were assessed at admission also took part in the follow-up evaluation conducted either at 30 days post-admission or at the time of discharge.

### 3.1 Sociodemographic characteristics of the patients

Table 1 presents the socio-demographic characteristics of the TBI patients. About half of the patients are between 18 to 44 years old. The mean age of the patients was 32.3 years, with a notable predominance of male patients. More than one-third respondents had no formal education. About one-sixth TBI patients were student. Among the working group, day-laborers had the highest percentage of TBI. Half of the female patients were housewives. Majority of the patients belonged to single family and a little less than half of the patients had a family income less than 128 USD [equivalents to 10 thousand Bangladeshi Taka (BDT)].

**Table 1:** Socio-demographic characteristics of the traumatic brain injury (TBI) patients at a tertiary care teaching hospital of Bangladesh (*N* = 659).

Variable	Frequency	Percent
Age		
<5 years	29	4.4
5–17 years	131	19.9
18–29 years	150	22.8
30–44 years	172	26.1
45–59 years	60	9.1
60 + years	117	17.7
Sex		
Male	533	80.1
Female	126	19.9
Education		
No Literacy	243	36.9
Primary	184	27.9
Secondary	168	25.5
Tertiary	62	9.4
Occupation		
Farmer	69	10.5
Business	83	12.6
Day laborer	96	14.6
Service	69	10.5
Driver (rickshaw/van/taxi/CNG/car/bus)	38	5.8
Student	112	17
Housewife	66	10
Retired/unemployed	36	5.4
Not applicable/child less than 6 years	49	7.4
Others	41	6.2
Family type		
Single family	475	72.1
Joint family	184	27.9
Family income in taka		
Less than 128 USD (10 thousand BDT)	175	48.2
More than 128 USD (10 thousand BDT)	188	51.8

### 3.2 Mechanism of traumatic brain injury

**Figure 1:**
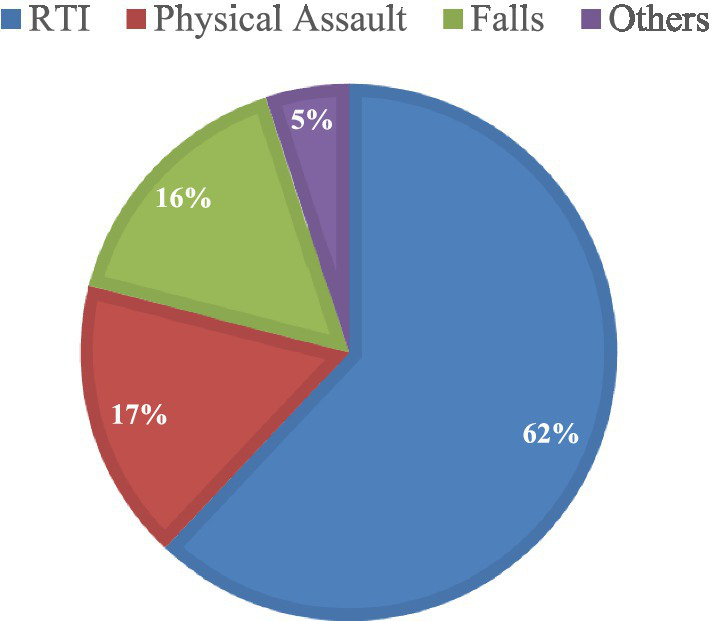
Events that led to TBI among the patients of a tertiary care teaching hospital in Bangladesh.

Figure 1 demonstrates the causes of traumatic brain injury among the respondents. Road traffic Injury was the leading event that led to TBI and more than half of the respondents experienced TBI following a road traffic crash. Physical assault was found to be the second leading cause for TBI and around one-sixth respondents were injured as a result of intentional violence by others. Furthermore, about 16% respondents experienced TBI due to fall from any level. Other causes of TBI included injuries from industrial machineries, injuries from home appliances, and getting hit by any animal.

Almost all (94.8) of the RTI incidents occurred in the main road. Pedestrians are the majority (43.8) among the respondents who experienced TBI due to road crashes, followed by drivers (28.3%). Furthermore, more than one-fourth (27.1%) RTI related TBI occurred due to motorcycle injury. Of all the fall injuries, fall from height was predominant (74.4) which included fall from rooftop (34.1%), fall from trees (23.2%), and fall during construction work (17.1%). Apart from main road (58.8%), other places for occurrence of TBI included yard and neighborhood (18.4%), home (7.2%), footpath (6.8%), and workplaces (6.5%).

### 3.3 Pre-hospital care and post-injury response

Table 2 presents the factors that describes post-injury response and pre-hospital care scenario following TBI. Majority (92.1%) of the patients did not receive any first aid treatment or CPR immediately after the injury. Pedestrians and family members are predominant among the persons who first attended the injured patients. Most (93.5%) of the patients were not immobilized while transferring from injury site to a health facility. Furthermore, a large number (82.4) of patients were also not immobilized while getting transferred from one health facility to another. About two-third of the patients sought treatment to nearby primary and secondary healthcare facilities after sustaining the injury. Only 18% patients came directly to this tertiary care health facility (DMCH). Subsequently, more than one-third (34.3%) of patients reported not receiving any treatment for their injury at the first level of contact with a health facility. Moreover, about half of the patients had 1 to 8 h gap in initiation of the treatment from the occurrence of the injury. In addition, a substantial (46.9%) number of patients had more than 8 h of delay between the incidence of injury and the initiation of treatment. All patients reached the health facility by their personally hired ambulances or vehicles. Almost all (97%) of the respondents reported of not using any safety measures such as using helmet, seatbelt, footpath, foot over-bridge, gripping aid, anti-slip mat, padded guards etc. at the time of injury.

**Table 2:** Pre-hospital care and post-injury response factors following traumatic brain injury among the patients of a tertiary care teaching-hospital of Bangladesh (*N* = 659).

Variables	Frequency	Percentage
Safety measure taken		
Yes	20	3
No	639	97
First attending person after injury		
Family member	243	36.9
Colleague/Friends	98	14.9
Pedestrian	257	39
Community people	61	9.2
Received first aid or CPR		
Yes	52	7.9
No	607	92.1
Immobilized during transfer from injury site to health center		
Yes	43	6.5
No	616	93.5
Immobilized during transfer from Health Centre to health center		
Yes	116	17.6
No	543	82.4
First level of contact for treatment		
Primary level facility	228	34.5
Secondary level facility	206	31.2
Tertiary level facility/DMCH	119	18.1
Private practitioner/private clinic	81	12.3
Non-registered practitioner	25	3.9
Received treatment from first health facility		
Yes	433	65.7
No	226	34.3
Time gap between occurrence of injury and initiation of treatment		
Less than 8 h	9	1.4
1 to 8 h	341	51.7
More than 8 h	309	46.9

### 3.4 Health outcome following TBI

The mortality due to traumatic brain injury (TBI) in this cohort was 10.3% (*n* = 68), with a calculated mortality rate of 3.6 deaths per 1,000 person-days. Approximately 30% of TBI patients presented with associated injuries, with limb injuries being the most common at 68.5%. Other associated injuries included spinal, thoracic, abdominal, and facial injuries. Following admission, nearly one-fourth (24.3%) of the patients required surgical intervention, which encompassed procedures such as craniotomy, decompression, surgical toileting, burr hole drainage, elevation of depressed fractures, and debridement.

**Figure 2:**
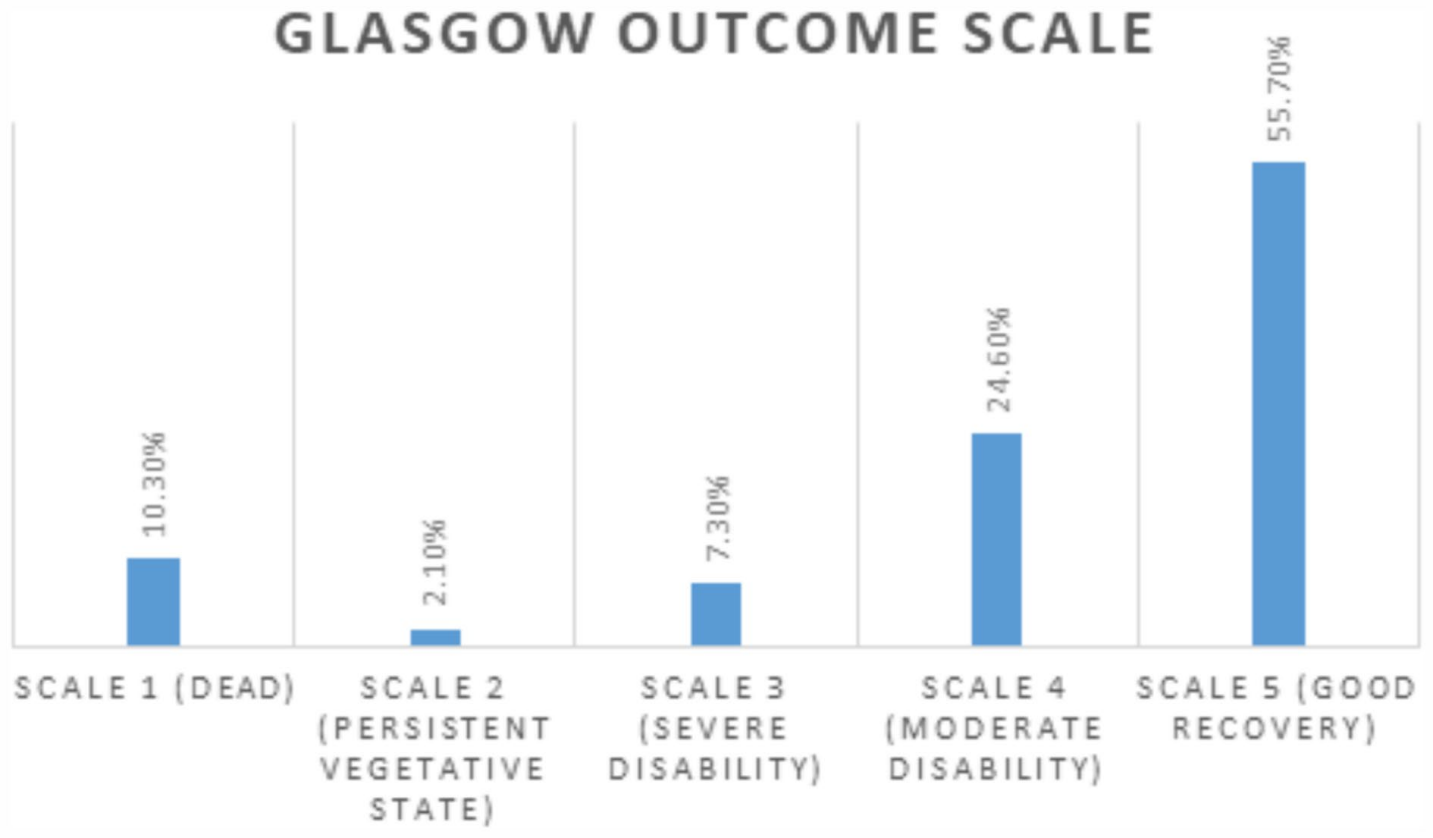
Distribution of Glasgow Outcome Scale (GOS) Outcomes among TBI Patients in a Tertiary Care Teaching Hospital in Bangladesh.

While more than half of the patients demonstrated good recovery, approximately one-fifth (19.5%) were classified as having poor outcomes on the Glasgow Outcome Scale (GOS) (Figure 2). Poor outcomes were considered as GOS scores ranging from 1 to 3, indicating death, a vegetative state, or dependence on others for daily activities. Furthermore, about one-third of patients reported experiencing some to severe problems across all domains of the EuroQol-5D-3L, with over 25% reporting severe issues related to mobility and pain (Supplementary Table S1).

### 3.5 RTI-related TBI: vulnerability and risk factors

As majority of the TBI occurred as a result of RTI, the vulnerability of RTI-related TBI was determined by the severity of injury and its consequences. Table 3 demonstrates the severity of RTI-related TBI at admission in comparison to TBI due to other causes. Respondents who sustained TBI due to RTI had 1.9 (95% CI: 1.2–3.1) times higher chance of falling into severe category of GCS score (<8) than respondents who had the injury due to any other causes. Furthermore, patients with RTI-related TBI were 1.6 (95% CI: 1.1–2.5) times more likely to suffer from >24 h’ duration of loss of consciousness than patients with TBI from other causes. Moreover, patients who endured TBI from road crashes had 1.4 (95% CI: 1.03–2.0) times higher chance of having associated injuries than patients who experienced TBI from non-RTI causes.

**Table 3:** Comparison of condition at admission between traumatic brain injury (TBI) patients due to road traffic injury (RTI) and TBI patients due to other causes.

Variables	Cause of TBI		OR	95% CI
	RTI, *N* (%)	Non-RTI, *N* (%)		
Glasgow Coma Scale (*N* = 659)				
Severe	78 (18.8)	26 (10.6)	1.9**	1.2–3.1
Mild to Moderate	336 (81.2)	219 (89.4)		
Duration of loss of consciousness (*N* = 654)				
More than 24 h	84 (20.5)	33 (13.5)	1.6*	1.1–2.5
Less than 24 h	326 (79.5)	211 (86.5)		
Post traumatic amnesia (*N* = 659)				
Yes	64 (15.5)	39 (16)	0.9	0.6–1.4
No	350 (84.5)	206 (84)		
Associated injury (*N* = 659)				
Yes	137 (33.1)	62 (25.3)	1.4*	1.03–2.0
No	277 (66.9)	183 (74.7)		

Bold values indicate statistically significant association. **p value = <0.01; *p value = <0.05.

RTI-related TBI also demonstrated significant adverse outcomes at follow-up compared to TBI resulting from other causes. About 55% of the patients who needed surgical intervention had RTI-related TBI. Mortality among patients with RTI-related TBI is 3 times (95% CI: 1.5–5.7) higher than patients with TBI due to other causes. Patients with RTI-related TBI also has 1.9 times (95% CI: 1.2–2.9) more chance of having poor outcome in GOS than patients with TBI from other causes. Further, comparison on EQ-5D-3L measurement revealed patients who had TBI from road crashes were 1.7 times (95% CI: 1.2–2.5) more likely to suffer from post-traumatic anxiety than patients who experienced TBI from non-RTI causes (Table 4).

**Table 4:** Outcome of traumatic brain injury (TBI) patients due to road traffic injury in comparison with TBI patients due to other causes at a tertiary care teaching-hospital in Bangladesh (*N* = 659).

Outcome variables	Cause of TBI		OR	95% CI
	RTI, *N* (%)	Non-RTI, *N* (%)		
Mortality outcome				
Yes (Dead)	56 (13.5)	12 (4.9)	3.0**	1.5–5.7
No (Alive)	358 (86.5)	233 (95.1)		
GOS outcome				
Poor (dead, vegetative state, dependent)	96 (23.2)	33 (13.4)	1.9**	1.2–2.9
Good (independent and normal)	318 (76.8)	212 (87.6)		
EQ-5D-3L mobility				
Problem (some to severe)	163 (45.5)	101 (43.3)	0.9	0.6–1.2
No problem	198 (55.5)	132 (56.7)		
EQ-5D-3L self care				
Problem (Some to severe)	140 (39.1)	76 (32.6)	1.3	0.9–1.8
No problem	218 (60.9)	157 (67.3)		
EQ-5D-3L usual activity				
Problem (Some to severe)	156 (43.6)	84 (36)	1.3	0.9–1.9
No problem	202 (56.4)	149 (64)		
EQ-5D-3L pain				
Problem (Some to severe)	157 (43.9)	98 (42)	1.1	0.7–1.5
No problem	201 (56.1)	135 (58)		
EQ-5D-3L anxiety				
Problem (Some to severe)	139 (38.8)	62 (26.6)	1.7**	1.2–2.5
No problem	219 (61.2)	171 (73.4)		
Residual complication during discharge				
Yes	63 (17.6)	32 (13.7)	1.3	0.8–2.1
No	295 (82.4)	201 (86.3)		

Bold values indicate statistically significant association. **p value = <0.01; *p value = <0.05.

#### 3.5.1 Risk factors for mortality due to TBI

Association and relationship of socio-demographic and clinical characteristics with mortality due to RTI-related TBI was analyzed through chi-square test and logistic regression. Ch-square test found that patients with a GCS score of less than 8 and thus on severe category at admission, had about 10 times higher chance of mortality than patients with a GCS score between 9 to 15 (mild to moderate) on admission. Furthermore, mortality is 5.7 times higher among patients who had a > 1-h duration of loss of consciousness than patients with <1-h duration of consciousness loss. Additionally, the chance of mortality is about two times higher among patients who had >8-h’ time gap in initiation of treatment than those who received treatment in <8-h. All other socio-demographic, clinical, and preventive factors, such as gender, education, family income, duration of post traumatic amnesia, and use of safety measures, had no significant association with mortality (Supplementary Table S2).

Table 5 presents the results from logistic regression after controlling the effect of all covariates. From Table 5, the GCS score, duration of loss of consciousness (LOC), and delay in treatment initiation, was further proven to have a predictive value regarding mortality due to RTI-related TBI. Mortality was 8.1 (95% CI: 4.1–16.2) times higher among patients with a GCS score on severe level (<8) than patients with a GCS score on mild to moderate level (9–15). Mortality is also about 3 times (95% CI: 1.4–8.1) higher among patients with >1-h duration of loss consciousness than patients with LOC of <1-h duration. Furthermore, a delay of >8-h in initiation of treatment caused 2.7 (95% CI: 1.4–5.5) times higher mortality among patients than a delay in initiation of treatment of <8-h duration. Socio-demographic factors along with other variables are found to have no statistically significant impact on mortality due to RTI-related TBI from the regression analysis.

**Table 5:** Factors associated with the mortality among road traffic injury related traumatic brain injury patients at a tertiary care teaching hospital of Bangladesh (*N* = 414).

Adjusted regression model					
Variables	B	Sig (p)	aOR	CI	
				Lower	Upper
Gender (Female)			1.4	0.6	3.5
Male	0.38	0.39			
Education (Literate)			0.8	0.4	1.8
No formal education	0.12	0.73			
Family Income (More than 10 thousand BDT)			1.4	0.6	2.8
Less than 10 thousand BDT	0.34	0.34			
Age	0.01	0.19	1.0	0.9	1.0
Glasgow Coma Scale score (mild to moderate)			8.1**	4.1	16.2
Severe	2.10	0.00			
Duration of loss of consciousness (less than 1 h)			3.4**	1.4	8.1
More than 1 h	1.22	0.00			
Time gap in treatment (less than 8 h)			2.7**	1.4	5.5
More than 8 h	1.02	0.00			
Associated injury (No)			1.2	0.5	2.4
Yes	0.19	0.59			

Bold values indicate statistically significant association. **p value = <0.01.

## 4 Discussion

Like other low-and middle-income countries (LMICs), Bangladesh is highly prone to injuries, yet research on traumatic brain injury (TBI)—one of the most severe forms of injury—remains limited. Although road traffic injuries (RTIs) are widely recognized as a major cause of TBI, their implications have not been thoroughly explored in this context. This study addressed this gap by not only presenting the burden and characteristics of TBI in Bangladesh but also highlighting the vulnerability associated with RTI-related cases and the limitation in the pre-hospital care. The findings emphasize the need for considering RTI as a significant factor requiring aggressive management and urgent policy attention for preventive measures. Moreover, the study generated valuable evidence on predictive factors influencing TBI outcomes, that can contribute to the development of a priority management protocol tailored for this setting.

The findings provided insights into the epidemiology of TBI in Bangladesh. Males of productive age group have been identified as the most vulnerable group for traumatic brain injury in Bangladesh and the mean age of the patients was 32.3 years. As men, particularly from young group, are in general more exposed to outer world and involved in manual and risky labor in Bangladesh, they are more prone to TBI than females. Additionally, RTI was identified as the leading cause of TBI, followed by physical assault. There has been an alarming rise in the road accidents in Bangladesh in recent years. According to the Bangladesh Road Transport Authority, RTI resulted in 4139 reported deaths in 2019 (32), which justifies the role of RTI as the primary mechanism of traumatic brain injury in Bangladesh. Studies of other LMICs including Southeast Asian region share the similar findings as well (16, 17). Other hospital-based studies on TBI in Bangladesh reported on similar line where they identified RTI as the major cause, males as the predominant group, and most patients between 20–35 years of age (25–27). Likewise, a nationwide injury survey in Bangladesh shared that RTI was the most common etiology of TBI, and that most of the injured patients were male (18). Some previous studies of TBI from similar geographic region documented physical assault as the second leading cause for TBI (15, 17), while some others documented fall as the second leading cause (16, 18). This study, however, found almost equal percentage of physical assault (17%) and fall (16%) as the mechanism of injury for TBI after RTI. This study also found that the pedestrians were the most common victims of RTI and the most common vehicle involved in the injury was the motorcycles. Research from developed countries has also shown that pedestrians are at a higher risk of experiencing TBI during road crashes, with motorcycles identified as the most frequently involved vehicle and more likely to be associated with severe TBIs (33–36). While Bangladesh continues to strive for the effective enforcement of vehicle and passenger safety regulations, a multi-faceted problem such as pedestrian injury still remains under-focused, as expressed in this study.

RTI has not only been identified as the leading cause of TBI, but also as the most vulnerable mechanism in terms of severity of the injury and its consequences. Patients with TBI from road crashes were more likely to have severe GCS scores, longer loss of consciousness, and associated injuries compared to other TBI causes. RTI-related TBIs showed three times higher mortality and nearly double the likelihood of poor GOS outcomes and post-traumatic anxiety than non-RTI-related TBIs. Studies from other LMICs have similarly found that the majority of severe TBI cases, as indicated by GCS scores, were caused by RTIs, with a higher percentage of mortality and morbidity observed among these patients (37, 38). The study identified key predictors of mortality for RTI-related TBI: a GCS score below 8, loss of consciousness lasting more than one hour, and a delay of over eight hours in initiating treatment. TBI patients with a GCS score below 8 had an eightfold higher risk of mortality compared to those with scores of 9–15. Additionally, patients with more than one hour of consciousness loss and treatment delays exceeding eight hours had about threefold increased risk of mortality. The only prior hospital-based cross-sectional study in Bangladesh that examined factors influencing prognosis reported similar findings, identifying GCS score, duration of unconsciousness, and treatment delay as significant predictive factors (25). Many other studies have also found GCS score as a predictive factor of mortality following TBI (15, 37, 39). Studies also identified length of coma or loss of consciousness as an early predictor of mortality due to TBI (33, 34). These clinical presentations with early predictive value for mortality are crucial for selection and timely management of the most vulnerable TBI patients.

The majority of the RTI-related TBI patients were unable to reach to a health facility within 1 h of the injury. In addition, a little less than half of the patients had a time gap of >8-h between the occurrence of injury and the initiation of treatment. This time gap since injury contributed to mortality of the TBI patients as mentioned above. This factor along with some other pre-hospital care factors explored from this study depicts the post-injury scenario of Bangladesh. Nearly none of the patients had any first aid or CPR treatment immediately after the injury. None of the patients received emergency transportation, and most were not immobilized during private transport. Two-thirds initially went to primary or secondary facilities before being referred to the tertiary center, with one-third reporting no treatment at the first facility. All these findings combine reflect to the lack of a dedicated emergency management system in Bangladesh. This study strengthens the fact that primary and secondary level health facilities of Bangladesh are largely incapacitated to deal with emergency conditions. Along with inadequate emergency transport mechanism, there is inadequate system in place for prompt referral of emergency patients. In addition, the lack of trained first aid responders in community and inadequate knowledge on basic emergency response following injury among the community people is also visible from this study. These post-injury consequences have substantial impact on the outcome of the patients, as reported by some recent studies that emphasized on the pre-hospital care of TBI patients for achieving a favorable outcome. Two review articles on TBI and a cross-sectional study at an Indian trauma center documented that a comprehensive pre-hospital care system including early transportation, first aid, and oxygenation results in improved outcome among patients (10, 21, 39). Researches in similar settings across South Asian region also signifies the need for a functional pre-hospital care and emergency management system in developing countries for a comprehensive management of TBI (17, 40–42). This underscores the importance of preventive measures in TBI management, highlighting the critical role of strengthening pre-hospital care and emergency support in reducing mortality.

Building on the critical gaps identified in pre-hospital care and emergency response for TBI in Bangladesh, the findings of this study also have the potential to contribute to the global TBI research agenda by highlighting the burden and predictors of outcomes in a low-resource setting, with a particular focus on the vulnerability of RTI-related TBI cases. The findings support and can inform global initiatives such as the World Health Organization (WHO)'s recently launched ‘Network for Global Emergency Care’ (43) and the ‘Global Emergency and Trauma Care Initiative (GETI) (44), which aim to specifically assist LMICs in evaluating their national emergency care systems, identifying deficiencies, and addressing disparities in TBI care and outcomes through collaborative, multi-center studies and unified approaches. Furthermore, research in developed countries, such as the CENTER-TBI (45) initiative in Europe, the TRACK-TBI study (46) in the United States, and the AUS-TBI study (47) in Australia, has underscored the importance of collecting detailed data to identify key predictors of TBI outcomes, plan emergency responses, and establish standardized care protocols. This study aligns with these global efforts by emphasizing history of RTI, GCS scores, treatment delays, and loss of consciousness as critical predictors of mortality. However, unlike high-resource settings where robust pre-hospital care systems and comprehensive trauma care are more accessible, the absence of emergency response mechanisms in Bangladesh highlights a significant gap in LMICs. Along similar line, studies in South Asian region, such as India, Pakistan, and Nepal have identified RTIs as a leading mechanism of TBI and emphasized pre-hospital care and timely intervention as essential factors for improving outcomes (40–42). The relevance of these findings, thus, extends to other LMICs where RTIs remain a predominant cause of TBI. Lastly, this study provides actionable evidence to guide policies aimed at improving emergency transport, first aid, and referral systems in resource-limited settings. It also reinforces the importance of implementing multi-center studies in LMICs to capture regional variations in TBI outcomes and to design locally tailored, yet globally informed, strategies to reduce the TBI burden.

### 4.1 Strengths and limitations

This study has several strengths. It is one of the few studies in Bangladesh that adopts a prospective approach to assess outcomes of traumatic brain injury (TBI), providing a detailed epidemiological analysis and insight into pre-hospital care. The findings highlight the risk factors for the most vulnerable TBI patients and add to the existing evidence on TBI prognosis in the country. By identifying predictive factors such as GCS score, duration of consciousness loss, and treatment delays, the study reinforces the significance of road traffic injuries (RTI) as a major cause of TBI. This contributes valuable evidence for policy-making in the prevention, management, and emergency care of TBI in Bangladesh. Additionally, the study’s setting in Dhaka Medical College and Hospital (DMCH)—the largest tertiary care hospital in Bangladesh—ensures that the patient sample represents diverse socio-economic backgrounds and regions, enhancing the relevance of the findings for a broader national context.

However, the study has some limitations. As a single-center, hospital-based study, the findings are not fully generalizable to the broader community. The lack of a uniform follow-up period due to the varying discharge times (before the 30-day follow-up) introduces potential bias in outcome assessment, though mortality rates were calculated using person-time data to mitigate this. Advanced statistical methods could have provided more robust analyses of the mortality predictors, addressing the limitation in follow-up method. Additionally, the study did not account for the impact of comorbid conditions on patient outcomes, which could be a significant factor.

We took several measures to limit potential sources of bias in this study. Selection bias was minimized by recruiting all eligible TBI patients consecutively during the study period at Dhaka Medical College and Hospital, ensuring comprehensive coverage of cases. However, the hospital-based nature of the study may still limit its generalizability to the broader population, particularly to those who did not seek hospital care. To mitigate information bias, data collection was standardized using a semi-structured questionnaire administered by trained medical practitioners. Validated tools such as the GCS, GOS, and EuroQol-5D-3L were employed to ensure consistent and reliable measurement of clinical outcomes. Efforts were also made to address recall bias, especially for variables like pre-hospital care and treatment delays, by collecting data at the point of admission and as soon as feasible. Additionally, multivariable logistic regression analysis was used to control for confounding factors, such as age, sex, and injury severity, when assessing the predictors of mortality and vulnerability associated with RTI-related TBI. Despite these efforts, the reliance on self-reported pre-hospital care data and the variability in the follow-up period may introduce residual bias, which we acknowledge as a limitation of the study.

Future research should consider a larger, community-based sample to assess the prevalence of TBI and outcomes for those who do not access tertiary care. Long-term outcomes, the influence of comorbidities, the quality of care provided at hospitals, and the economic burden of TBI at both individual and institutional levels should also be explored to broaden the understanding and improve TBI management strategies in low-resource settings like Bangladesh. Future studies could also incorporate trauma scoring systems to assess the impact of associated injuries or multiple traumas on TBI outcomes, which was beyond the scope of this study.

## 5 Conclusion

RTI remains the most critical mechanism of TBI, disproportionately affecting young males in the economically productive age group. This demographic vulnerability underscores the need for targeted policy measures, such as stricter enforcement of road safety laws, awareness campaigns for high-risk groups, and investment in infrastructure to mitigate RTIs. The study also identified key clinical predictors of mortality, including GCS and duration of loss of consciousness, which can aid in the initial triage of TBI patients and facilitate timely management for severe cases within healthcare facilities. Moreover, the findings highlight significant gaps in pre-hospital care and emergency response in Bangladesh, including delayed treatment initiation, lack of community-level emergency response knowledge, and insufficient training and logistics for first responders. To address these barriers, we recommend implementing community-based education programs on basic first aid and emergency response, establishing a coordinated pre-hospital care system with trained responders, and improving referral mechanisms for timely care. Policymakers are urged to prioritize the development of a comprehensive emergency management framework that integrates pre-hospital care, rapid transport, and early intervention strategies. Additionally, this study emphasizes the importance of multi-sectoral collaboration and multi-center research to contextualize findings and design locally adaptable, evidence-based interventions aimed at reducing the burden of TBI in Bangladesh and similar low-resource settings.

## Data Availability

The original contributions presented in the study are included in the article/Supplementary material, further inquiries can be directed to the corresponding author.
