# Supplementary material for: Epidemiology, outcomes, and risk factors of traumatic brain injury in Bangladesh: a prospective cohort study with a focus on road traffic injury-related vulnerability
Source: Front Public Health. 2025 Feb 14;13:1514011. doi: 10.3389/fpubh.2025.1514011 (PMC11868135; doi:10.3389/fpubh.2025.1514011)
Supplement: Supplementary file 1 [file Table_1.DOCX]

Supplementary Material

Title: Epidemiology, Outcomes, and Risk Factors of Traumatic Brain Injury in Bangladesh: A Prospective Cohort Study with a Focus on Road Traffic Injury-Related Vulnerability

*Farah Naz Rahman, Sukriti Das, Manzur Kader, Saidur Rahman Mashreky*

Supp. Table 1: EQ-5D-3L Domain Response among TBI patients during follow-up

| Domain | No Problem (%) | Some Problem (%) | Severe Problem (%) |
| --- | --- | --- | --- |
| Mobility | 59.9 | 10.8 | 29.1 |
| Self-care | 67.2 | 21.3 | 11.4 |
| Usual Activity | 63.5 | 20.2 | 16.1 |
| Pain | 61.3 | 12.0 | 26.6 |
| Anxiety/Depression | 69. | 12.9 | 17.6 |

**Supp.** **Table-2: Factors associated with the mortality among Road Traffic Injury related Traumatic Brain Injury patients at a tertiary care teaching hospital of Bangladesh.**

| **Variables** | **Outcome** | | **OR** | **95% CI** |
| --- | --- | --- | --- | --- |
|  | **Dead**, N (%) | **Alive**, N (%) |  |  |
| **Glasgow Coma Scale** | | | | |
| Severe (<8) | 33 | 45 | **9.98**** | 5.38-18.5 |
| Mild to Moderate (9-15) | 23 | 313 |  |  |
| **Duration of loss of consciousness** | | | | |
| More than 1 hour | 47 | 169 | **5.71**** | 2.72-12.02 |
| Less than 1 hour | 9 | 185 |  |  |
| **Post Traumatic Amnesia** | | | | |
| Yes | 4 | 60 | 0.38 | 0.13-1.09 |
| No | 52 | 298 |  |  |
| **Associated Injury** | | | | |
| Yes | 24 | 111 | 1.67 | 0.93-2.96 |
| No | 32 | 247 |  |  |
| **Time Gap in Initiation of Treatment** | | | | |
| >8 hours | 35 | 154 | **2.21**** | 1.24-3.94 |
| <8 hours | 21 | 204 |  |  |
| **Safety Measures** | | | | |
| Yes | 1 | 17 | 2.74 | 0.36-21.02 |
| No | 55 | 341 |  |  |
| **Gender** | | | | |
| Male | 47 | 287 | 1.29 | 0.60-2.76 |
| Female | 9 | 71 |  |  |
| **Education** | | | | |
| No literacy | 21 | 119 | 1.21 | 0.67-2.16 |
| Literate | 35 | 239 |  |  |
| **Family Income** (in BDT) | | | | |
| Less than 10 thousand | 20 | 91 | 1.78 | 0.99-3.18 |
| More than 10 thousand | 36 | 267 |  |  |

**Study Questionnaire**

| **A. Patient Information:** | |
| --- | --- |
| A1. Age of the patient (In years) |  |
| A2. Gender of the patient (Enumerator will choose the response based on visual observation)  1. Male, 2. Female, 3. Transgender |  |
| A3. Religion of the patient  1. Islam, 2. Hinduism, 3. Christianity, 4. Buddhism, 5. Others, specify_____________________________________________ |  |
| A5. Educational Status of the patient  Number of completed educational years |  |
| A6. Occupation of the patient  1, Farmer \| 2, Business \| 3, Student \| 4, Service \| 5, Housewife \| 6, Retired \| 7, Unemployed \| 8, Day-laborer \| 9, Servant \| 10, Rickshaw puller \| 11, Van driver \| 12, Taxi/CNG auto rickshaw/Bus driver \| 13, Private car driver \| 14, Beggar \| 15, Not applicable (child less than 6 years) \| 98, I don’t know \| 99, Others, specify ____________________________________ |  |
| A7. What is his/her family type?  1. Single family, 2. Joint family, 3. Lives alone, 98. I don’t know |  |
| A8. How many family members does he/she have including him/her? |  |
| A9. How many earning members are there in his/her families except him/her? |  |
| A10. What is the approximate monthly family income of the patient? (in Taka) |  |
| A11. What is the approximate monthly income of the patient? |  |
| A12. What is the approximate monthly expenditure of the patient’s family? |  |

| **B. Factors for TBI:** | |
| --- | --- |
| B1.What was the nature of injury in this patient?  1. Intentional (by another person), 2. Intentional (self-harm), 3. Non-intentional, 98. I don’t know, 99. Others, specify_____________________ |  |
| B2. What was the event that led to TBI in this patient?  1. Fall from height, 2. Slipped or tripped and fell down, 3. Road traffic accident (RTA), 4. Hit by an animal, 5. Physical assault, 6. Injured by industrial machinery, 7. Injured by medical intervention, 8. Sports related event, 9. Injured by home appliances, 10. Shaking the child vigorously, 98. I don’t know, 99. Others, Specify_____________________________________________________ |  |
| B3. If RTA, which of the following was the role played by the patient?  1. Driver, 2. Passenger, 3. Pedestrian, 98. I don’t know |  |
| B3a. During RTA, which vehicle was the patient in (if driver or passenger) /hit by (if pedestrian)?  1. Motor car, 2. Motor bike, 3. Bus, 4. Three wheeler, 98. I don’t know, 99. Others, specify ____________________________________________ |  |
| B4. If sports injury, Which sport was the patient involved in?  1. Cricket, 2. Football, 98. I don’t know, 99. Others, specify__________________________________________________ |  |
| B5. If the event of injury was fall from height, which was the following event happened?  1. Fall form tree, 2. Fall from rooftop (home), 3. Fall during construction work, 4. Fall during electric/cable repairing, 98. I don’t know, 99. Others, specify ___________________________________________________ |  |
| B5a. If fall from tree, which tree the patient fell from?  1. Mango, 2. Black berry, 3.Lichee, 98. I don’t know, 99. Others, specify______________________________________________ |  |
| B6. What was the object causing injury (if applicable)  1. Blunt object, 2. Sharp object, 3. Bullet, 98. I don’t know, 99. Others, specify ______________________________________________ |  |
| B7. What was the place where the injury happened?  1. Kitchen, 2. Bathroom, 3. Other room of a house except kitchen & bathroom, 4. Staircase, 5. School room, 6. Office room, 7. Office-open area, 8. Industrial area, 9. Recreational area, 10. Children's playground, 11. Road-footpath, 12. Road (main), 13. Farm, 14. Countryside, 15. Medical service area, 98. I don't know, 99. Others, specify___________________ |  |

| **C. Application of safety measure** | |
| --- | --- |
| C1. Was he/she maintaining any safety measure (maintain a precaution that could have prevented the injury) during the time of injury?  1. Yes, 2. No, 98. I don’t know |  |
| C1a. If yes, then which safety measure he/she was using? (Multiple ans.)  1. Helmet, 2. Seat belt, 3. Using footpath, 4. Using foot over bridge, 5. Using anti-slip mat, 6. Using Padded guards, 7. Using gripping aid, 8. Using measures to prevent electric shock, 98. I don’t know, 99. Others, specify__________________________________________ |  |

| **D. Pre-hospital care** | |
| --- | --- |
| D1. Was there any person around at the time of injury?  1. Yes, 2. No, 98. I don’t know |  |
| D1a. If yes, then who? (multiple response)  1. Family member, 2. Colleague, 3. Friend, 4. Pedestrian, 5. Other farmers, 6. Villagers, 7. Neighbor, 98. I don’t know, 99. Others, specify______________________________ |  |
| D2. Was he or she given any first aid or CPR after injury?  1. Yes, 2. No, 98. I don’t know |  |
| D3. Was he or she immobilized during transfer from accident site till hospital admission?  1. Yes, 2. No, 98. I don’t know |  |
| D4. If answer, of D2 or D3 is yes, was the person who helped at that time trained in giving first aid or CPR?  1. Yes, 2. No, 98. I don’t know |  |
| D5. Where was the patient taken first after injury?  1. Home, 2. Health facility, 3. Nowhere, 98. I don’t know |  |
| D6, If taken to health facility then where?  1. Government sub-center, 2. Community clinic, 3. Health center, 4. Private health facility, 98. I don’t know, 99. Others, specify______________________________________________ |  |
| D7. Who first attended the patient after injury?  1. Registered physician, 2. Health assistant/visitor/family welfare assistant, 3. Quack doctor, 4. Traditional healer/Kabiraj, 5. Religious Healer, 6. Homoeopathic practitioner, 7. Pharmacy/Drug seller, 8. Village doctor, 98. I don’t know, 99. Others, specify________________________________ |  |
| D8. If he/she was not taken to hospital immediately after the injury, then, what was the cause behind it? (multiple response)  1. There was no one around to take to the hospital, 2. It didn’t seem necessary, 3. No one around patient knew that hospitalization was necessary, 4. Financial problem, 5. Transportation problem, 98. I don’t know, 99. Others, specify____________________________________________ |  |
| D9. What was the approximate time gap between injury and contact with a registered physician? (in hours) |  |
| D10. What was the approximate time gap between injury and starting of appropriate treatment? (in hours) |  |

D11. Fill up the following table in consecutive order of time (start from the place where the patient was first taken for treatment)

| Place where taken for treatment | Date | Time | Receiving treatment | Reason for choosing the place for treatment | Person who made decision regarding place of treatment |
| --- | --- | --- | --- | --- | --- |
|  |  |  |  |  |  |
|  |  |  |  |  |  |
|  |  |  |  |  |  |
|  |  |  |  |  |  |
|  |  |  |  |  |  |

***Codes for question D11***

Place where taken for treatment

1. Private medical practitioner, 2.Quack doctor, 3.Traditional healer/Kabiraj, 4.Religious Healer, 5.Homoeopathic practitioner, 6.Pharmacy/Drug seller, 7.Village doctor, 8.Health assistant/visitor/family welfare assistant, 9.Upazila health complex, 10.District hospital, 11.Medical college hospital, 12.Private clinic/hospital, 98.I don’t know, 99. Others, specify

Receiving treatment

1. Yes, 2.No, 98.I don’t know

Reason for choosing the place for treatment

1. Referred from the previous hospital/service provider, 2.Close to home, 3.Close from incident site, 4.Lesser cost for treatment, 5.dissatisfied from previous service provider, 98. I don’t know, 99. Others, specify

Person who made decision regarding place of treatment

1. Self, 2.Family member, 3.Relative, 4.Community people, 5.previous service provider, 98. I don’t know, 99.Others specify

| **E. Characteristics of TBI (This section is to be filled up by using patient’s diagnosis & treatment records)** | |
| --- | --- |
| E1. Is the state of the patient at the time of admission?  1.Unconscious, 2.Conscious |  |
| E2. What was the GCS scale of the patient on admission?  1.3, 2.4, 3.5, 4.6, 5.7, 6.8, 7.9, 8.10, 9.11, 10. 12, 11.13, 12.14, 13.15 |  |
| E3. What was the duration of loss of consciousness in this patient?  1. Few seconds to minutes, 2. 1-24 hours, 3. More than 24 hours |  |
| E4. What was the duration of post-traumatic amnesia (PTA) in this patient?  1. Less than 1 hour, 2. 1-24 hours, 3. More than 24 hours |  |
| E5. Which of the following associated physical complications of TBI has developed in the patient after injury? (multiple response)  1. Vegetative state, 2. Amnesia, 3. Post traumatic seizure, 4. Cranial nerve neuropathy, 5. Post-concussion syndrome, 6. Meningitis, 7, Brain abscess, 8. Cognitive deficit, 9. Communication problem, 10. Sensory deficit, 11. Emotional and behavioral problem, 12. Deep vein thrombosis, 13. Pulmonary embolism, 14. Pneumonia, 15. Pressure sore, 16. Progressive multiple organ failure, 17. Hydrocephalus, 18. Raised intracranial pressure, 19. Hypertension, 20. Low blood pressure, 21. Gastrointestinal disturbance, 22. UTI, 23. Spasticity, 99.Other,specify_____________________________________________________________________________________________________ |  |
| E6. Are there any other injuries beside TBI in this patient?  1.Yes, 2.No |  |
| E6a. If yes, what is/are those injuries? (multiple response)  1. Head injury, 2.Thoracic injury, 3. Abdominal injury, 4. Limb injury, 99.Others, specify_____________________________________ |  |
| E7. What was the required treatment modality for this patient?  1.Conservative, 2.Surgical |  |
| E8. Which of the following conservative management was received by this patient? (multiple response)  1. Resuscitation, 2. Mannitol, 3. Steroids, 4. Antibiotics, 5. Anticonvulsants |  |
| E9. If surgical, how many surgeries were required for the patient till discharge? |  |
| E9a. What type of surgery was needed? (multiple response)  1, Debridement \| 2, Elevation of depressed fracture \| 3, Surgical toileting \| 4, Craniotomy \| 5, Decompressive craniectomy \| 6, Decompression with or without removal of blood \| 7, Burr hole |  |

**F. Economic burden during hospital stay**

F1. Hospital admission related cost of the patient (In Taka)

| **a)Transport** | **b)Investigations** | **c)Medicines** | **d)Surgical** | **e)Others** |
| --- | --- | --- | --- | --- |
|  |  |  |  |  |

F2. Indirect costs associated with the TBI (In Taka)

| **a)Patient’s food and other necessity related cost** | **b)Patients attendants’ food and other costs due to hospital stay** | **c)Patients attendants’ transportation cost** | **d)Cost for property loss** | **e)Cost for loss of patient’s working days due to hospital stay** | **f)Cost for loss of attendant’s working days due to hospital stay** |
| --- | --- | --- | --- | --- | --- |
|  |  |  |  |  |  |

| **G. Health outcome at time of discharge (G1-G3 numbered questions are to be filled up by using patient’s diagnosis & treatment records)** | |
| --- | --- |
| G1. Is the state of the patient at the time of discharge?  1.Dead, 2.Unconscious, 3.Conscious |  |
| G2. What is the condition of the patient according to Glasgow coma outcome score?  1, Death \| 2, Vegetative state\| 3, Lower severe disability \| 4, Upper severe disability \| 5, Lower moderate disability \| 6, Upper moderate disability \| 7, Lower good recovery \| 8, Upper good recovery |  |
| G3. At time of discharge, what is the disability category of the patient according to Disability rating scale (DRS)?  1. Mild, 2. Partial, 3. Moderate, 4. Moderately severe, 5. Severe, 6. Extremely severe, 7. Vegetative state, 8. Extreme vegetative state |  |
| G4. Is there any residual complication in this patient at the time of discharge?  1. Yes, 2. No |  |
| G4a. If yes, that what is/are the complication/s? (multiple response)  1. Coma,2. Stupor, 3. Persistent vegetative state, 4. Minimally conscious state, 5. Locked in syndrome, 6. Brain death 7. Seizures, 8. Post traumatic epilepsy, 9. Stroke, 10. Damaged cranial nerve/s, 11. Post-concussion syndrome 12. Intracranial hemorrhage, 13. Pituitary damage, 14. Meningitis, 15. Brain abscess, 16. Cognitive deficit, 17. Communication problem, 18. Sensory deficit, 19. Emotional and behavioral problem, 20. Deep vein thrombosis, 21. Pulmonary embolism, 22. Pneumonia, 23. Pressure sore, 24. Progressive multiple organ failure, 25. Hydrocephalus, 26. Raised intracranial pressure, 27. Hypertension, 28. Low blood pressure, 29. Gastrointestinal disturbance, 30. Vascular complications, 31. Spasticity, 99.Other,specify___________________________________________________________ |  |
| G5. What is the height of the patient? |  |
| G6. What is the weight of the patient? |  |

G7. Fill up the following table for assessing Quality of Life of TBI patient (EQ-5D tool)

| **QUALITY OF LIFE TOOL**  **(For Patients of 16 years and above)** | | | | |
| --- | --- | --- | --- | --- |
| **EQ-5D Dimension** | **Level 1** | **Level 2** | **Level 3** |  |
|  | **1** | **2** | **3** |  |
| **a)Mobility** | No problem in walking | Some problem in walking | Confined to bed |  |
| **b)Self-care** | No problem | Some problem | Unable to wash or dress |  |
| **c)Usual activity** | No problem | Some problem | unable to perform my usual activities |  |
| **d)Pain/Discomfort** | No pain or discomfort | Moderate pain or discomfort | Extreme pain or discomfort |  |
| **e)Anxiety/Depression** | Not anxious or depressed | Moderately anxious or depressed | Extremely anxious or depressed |  |
| **G8. Self-rated health**(Best imaginable health state, 100 and Worst imaginable health state, 0; missing value 999) | | | |  |

**
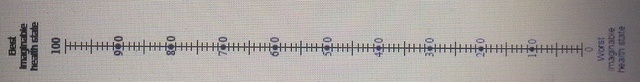
**

| **H. Rehabilitative management** | |
| --- | --- |
| H1. Was any rehabilitative management provided to the patient at the time of discharge?  1. Yes, 2. No |  |
| H1. If yes, then which management was advised or given? (multiple response)  1. Physical therapy, 2. Occupational therapy, 3.Therapy for issues of bowel and bladder dysfunction 4. The management of pressure ulcers, 5.Psychological therapy, 6. Speech-language therapy, 7. Others, specify_______________________________________________________ |  |
